# Highly Recurrent Copy Number Variations in *GABRB2* Associated With Schizophrenia and Premenstrual Dysphoric Disorder

**DOI:** 10.3389/fpsyt.2020.00572

**Published:** 2020-06-30

**Authors:** Ata Ullah, Xi Long, Wai-Kin Mat, Taobo Hu, Muhammad Ismail Khan, Li Hui, Xiangyang Zhang, Peng Sun, Mingzhou Gao, Jieqiong Wang, Haijun Wang, Xia Li, Wenjun Sun, Mingqi Qiao, Hong Xue

**Affiliations:** ^1^ Applied Genomics Center and State Key Laboratory of Molecular Neuroscience, Hong Kong University of Science and Technology, Division of Life Science, Hong Kong, Hong Kong; ^2^ Suzhou Guangji Hospital, The Affiliated Guangji Hospital of Soochow University, Suzhou, China; ^3^ Institute of Psychology, Chinese Academy of Sciences, Beijing, China; ^4^ School of Basic Medicine, Shandong University of Traditional Chinese Medicine, Jinan, China; ^5^ School of Basic Medicine and Clinical Pharmacy, China Pharmaceutical University, Nanjing, China

**Keywords:** GABAA receptors, schizophrenia, premenstrual dysphoric disorder, copy number variations, genetic factors

## Abstract

**Objective:**

Although single-nucleotide polymorphisms in *GABRB2*, the gene encoding for GABA_A_ receptors β2 subunit, have been associated with schizophrenia (SCZ), it is unknown whether there is any association of copy number variations (CNVs) in this gene with either SCZ or premenstrual dysphoric disorder (PMDD).

**Methods:**

In this study, the occurrences of the recurrent CNVs esv2730987 in Intron 6 and nsv1177513 in Exon 11 of *GABRB2* in Chinese and German SCZ, and Chinese PMDD patients were compared to controls of same ethnicity and gender by quantitative PCR (qPCR).

**Results:**

The results demonstrated that copy-number-gains were enriched in both SCZ and PMDD patients with significant odds ratios (OR). For combined-gender SCZ patients versus controls, about two-fold increases were observed in both ethnic groups at both esv2730987 (OR = 2.15, *p =* 5.32E−4 in Chinese group; OR = 2.79, *p =* 8.84E−3 in German group) and nsv1177513 (OR = 3.29, *p =* 1.28E−11 in Chinese group; OR = 2.44, *p =* 6.17E−5 in German group). The most significant copy-number-gains were observed in Chinese females at nsv1177513 (OR = 3.41), and German females at esv2730987 (OR=3.96). Copy-number-gains were also enriched in Chinese PMDD patients versus controls at esv2730987 (OR = 10.53, *p =* 4.34E−26) and nsv1177513 (OR = 2.39, *p =* 3.19E−5).

**Conclusion:**

These findings established for the first time the association of highly recurrent CNVs with SCZ and PMDD, suggesting the presence of an overlapping genetic basis with shared biomarkers for these two common psychiatric disorders.

## Introduction

Schizophrenia (SCZ) is a major psychiatric disease with a significant genetic component (1, 2). Among the genes that have been related to SCZ, the single-nucleotide polymorphisms (SNPs) of the GABA_A_ receptors β_2_ subunit gene (*GABRB2*) introns 8 and 9 in chromosome 5 were found by us to be associated with SCZ in multiple populations (3, 4), and the association was confirmed in a genome-wide linkage scan (5). These SCZ-associated SNPs, located in the vicinity of the AluYi6AH-151 insertion on *GABRB2* (6, 7), influenced genotype-dependent expression, and alternative splicing of the *GABRB2* transcript giving rise to a reduced long-to-short isoform ratio of the β_2_ subunit in SCZ brains (8, 9). The reduced ratio in turn rendered the β_2_ subunit-containing GABA_A_ receptors less susceptible to fatigue upon repeated stimulation, thereby retarding the turn-off of inhibitory activity of the neurons in the face of lowered energy status (9–12). As a result, some of the haplotypes in the region such as H26 and H73 have been identified as protective, while other haplotypes such as H19 and H81 have been identified as susceptibility-enhancing, toward the development of SCZ (7).

Besides the strong evidence from schizophrenic brains for an important role of *GABRB2* SNP in SCZ etiology, recently we have found that knock-out of either one or both copies of the *GABRB2* gene in mice also brought about a wide range of phenotypic alterations resembling the positive symptoms, negative symptoms and cognitive deficits of SCZ. Moreover, a range of theses SCZ-like phenotypic alterations in the knock-out mice could be ameliorated or reversed by the antipsychotic drug risperidone. These findings from the knock-out mice model were therefore in accord with those based on the SCZ-associated *GABRB2* SNPs and haplotypes in establishing a pivotal role of *GABRB2* in the origin of SCZ (13). In recent years, SCZ has been associated with a wide range of copy number variations (CNVs) outside of the *GABRB2* gene, pointing to the involvement of CNVs in some aspects of SCZ etiology (14). Accordingly, an objective of the present study was to determine whether any of the CNVs in *GABRB2* might be associated with SCZ.

Premenstrual dysphoric disorder (PMDD) is a severe form of premenstrual syndrome (PMS) that afflicts 5–10% of women in their reproductive years (15). Both animal and human studies were indicative of possible linkages of periods of neurosteroid fluctuation and alteration with the sensitivity of GABA_A_ receptors to neurosteroids, leading to mood instability in PMDD (16, 17). Moreover, PMDD has been associated with two different groups of sex hormone-related genes, *viz.*, the estrogen receptor alpha gene *ESR1* (18), and the ESC/E(Z) genes that affect how sex hormones interact with other genes (19), but genetic association of GABA_A_ receptors with PMDD has been largely lacking. Since the SCZ-associated SNPs in *GABRB2* have been associated with not only SCZ but also bipolar disorder (10), heroin addiction (20), and altruism (21), and some of the SCZ-associated CNVs have been associated with other mental disorders including autism and mental retardation (14), the present study was directed as well to the analysis of *GABRB2* CNVs in both SCZ and PMDD patients. The results obtained showed that the recurrent CNVs nsv1177513 in Exon 11 and esv2730987 in Intron 6 of *GABRB2* were associated with both SCZ and PMDD.

## Materials and Methods

### Participants

For the SCZ study, genomic DNA was prepared using the phenol-chloroform method from the blood samples of 285 Chinese SCZ patients (160 male and 125 female) with a mean age of 39.7 ± 12.7 years, from Beijing Hui Long Guan Hospital, and 286 controls (161 male and 125 female) with a mean age of 24.4 ± 9.2 years from Hong Kong Red Cross; and 207 German SCZ patients (101 male and 106 female) with a mean age of 31.3 ± 9.3 years, and 210 controls (110 male and 100 female) with a mean age of 29.3 ± 9 years from hospitals affiliated with the University of Wurzburg. SCZ (ICD-11: F20.9) is a poorly understood mental disorder that presents hallucinations, delusions, and other cognitive issues. Typically, it has three phases—prodromal, acute, and recovery. Since the prodromal phase is mostly reported for subjects aged 20–35, and our mean sample size was 39.5 ± 12.7 years, a majority of the subjects would belong to the acute phase.

PMDD (ICD-11: F32.81) is characterized by cognitive-affective and physical symptoms in the week before mensis, and it has been designated as a disorder by American Psychiatric Association. For the PMDD study, genomic DNA was prepared using the phenol-chloroform method from the blood samples of 215 Chinese PMDD women with a mean age of 24.5 ± 4.1 years and 208 controls with a mean age of 21.4 ± 2.1 years from Shandong University of Traditional Chinese Medicine Hospital, Jinan, People’s Republic of China. All samples were obtained with the written consent of subjects. The research was conducted with ethics approvals from the human participants’ research panel of the Hong Kong University and Science and Technology. PMDD sample collection was approved by China ethics committee of registering clinical trials (ID: ChiECRCT-2013030).

### Determination of Copy Number by Quantitative PCR

To monitor *GABRB2* CNVs in the peripheral white blood cell samples, two CNV regions with the highest recorded recurrence in the gene, *viz.*, nsv1177513 on Exon 11 (226 occurrences; chr5:160,715,688-160,717,804) and esv2730987 on Intron 6 (3 occurrences; chr5:160,796,703-160,797,020) were retrieved from the Database of Genomic Structural Variation (dbVar), along with the smaller CNV regions of esv2659732, esv3842929 and esv3077326 that overlapped with nsv1177513, and esv2667994 that overlapped with esv2730987 (see **Supplementary Table S1**). The copy number status at these regions in SCZ patients, PMDD women, and controls were determined by quantitative PCR (qPCR) using the LightCycler^®^ 480 SYBR Green I Master kit on the Light Cycler 480 platform (Roche) with primers designed based on the sequences of nsv1177513 and esv2730987, respectively. *RNase P* and *ALB* were employed as reference genes in qPCR, and all tests were run in triplicates. The qPCR reaction mix contained 5-µl Roche L480 SYBR Green (ROX Free), 0.2-µl 10 µM forward primer, 0.2-µl 10 µM reverse primer and 4.6-µl (4 ng/µl) DNA sample. The primer sequences for esv2730987 and nsv1177513 were designed using NCBI PrimerBlast (**Table 1**). The plates containing qPCR reaction mix in the wells were centrifuged and placed into LightCycler 480 qPCR. The PCR schedule consisted of 95°C for 5 min, 40 cycles of 95°C for 10 s, 60°C for 1 min, and 72°C for 30 s.

**Table 1:** Primer sequences for measuring copy number status by qPCR.

Gene (CNV)	Primer	Ref.
*GABRB2* (esv2730987)	Forward: 5′-CATAACAGGTTTGCTATATTTGCCA-3′	
	Reverse: 5′-GCCTTCACAAGTTAGATGCACA-3′	
*GABRB2* (nsv1177513)	Forward: 5′-TTCTGTTCACTCCTTTCTGGTTT-3′	
	Reverse: 5′-TTGTTCCTTTCAACCAAAGACTCC-3′	
*ALB*	Forward: 5′-AATGCTGCACAGAATCCTTGGT-3′	(22)
	Reverse: 5′-TCATCGACTTCCAGAGCTGAAA-3′	
*RNaseP*	Forward: 5′-CTAACAGGGCTCTCCCTGAG-3′	(23)
	Reverse: 5′-CAGCCATTGAACTCACTTCG-3′	

The threshold cycle number (Ct) was calculated for all the samples with LightCycler^®^ 480 SW 1.5.1 software. Samples with a Ct value more than 30, and samples with a difference between any two of the replicate measurements greater than 0.5, were excluded from further analysis (24). The target gene expression level was normalized with respect to *RNase P* and *ALB* expression. The difference between the Ct value of the target and the average Ct values of *RNase P* and *ALB* was recorded as ΔCt. One known control sample was included on each plate to monitor the batch to batch variation, and to serve as the calibrator required by the ΔΔCt method (25). The normalized fold expression of the target gene was calculated as 2^(−ΔΔCt), and the exact copy number was defined as 2*2^(−ΔΔCt) (24). A copy number that fell between 1.5 and 2.5 was regarded as CN-neutral (N), and a copy number above or below these thresholds was considered to be a CN-gain (G) or CN-loss (L), respectively. The copy numbers of esv2730987 and nsv1177513 determined for the SCZ patients, PMDD patients and controls were indicated in **Supplementary Tables S2A–I**.

### Statistical Analysis

All comparisons of CNV or CNV haplotype frequencies were conducted using Chi-square tests, and unadjusted *p* values were reported without multiple test correction. The 95% confidence interval (CI) of the odds ratio (OR) was estimated by

$$\mathrm{CI}=e^{\ln(OR)\pm 1.96\times \sqrt{\frac{1}{x1}+\frac{1}{x2}+\frac{1}{y1}+\frac{1}{y2}}},$$

where *x*1, *x*2, *y*1, and *y*2 were the four figures employed to calculate the OR. The heritability of liability for SCZ was estimated by percentage of familial risk (or logRR genetic variance) attributable to CN-gain at the risk loci using INDI-V online tool at http://cnsgenomics.com/shiny/INDI-V/ with a baseline population risk of disease of 1.0% and a sibling recurrence risk of 8.8 (26). The logRR values were 2.53% and 9.30% for CN-gain at esv2730987 and nsv1177513, respectively. The combined contribution of the two loci was estimated by collapsing the CN-gains at the two loci into a single signal, and a sample with a CN-gain at either one of these two loci was counted as one CN-gain.

## Results

### CNVs in Schizophrenic Cohorts of Chinese and German Origins

**Figure 1:**
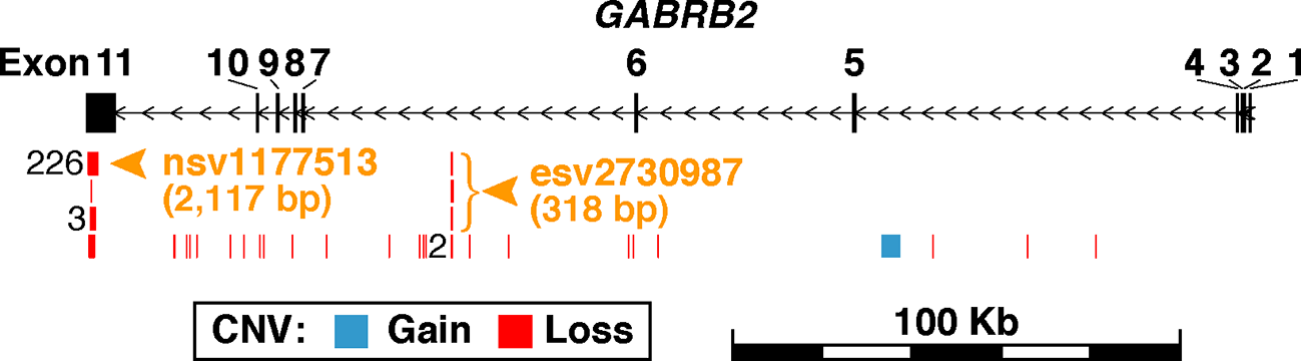
Schematic representation of germline CNVs in *GABRB2*. The region shown spans 20-Kb upstream and downstream of *GABRB2* at chr5:160,715,436-160,975,130. Exons are represented by the black vertical rectangles, and introns by horizontal line segments between the exons. Each exon is numbered above its corresponding rectangle. Black arrows indicate direction of transcription. CNVs retrieved from the database of human genomic structural variation (dbVar; file date 2018-03-04) are shown in blue for copy-number-gains and red for copy-number-losses. CNVs with identical genomic coordinates are merged as one in the figure, with their number of occurrences labeled left to their positions. A total of 261 CNVs falling within *GABRB2* are mapped to 31 parental copy-number-regions in dbVar (see the full list of CNVs in **Supplementary Table S1A** and the summary statistics of their parental copy-number-regions in **Supplementary Table S1B**). CNVs are found to be highly recurrent in Exon 11 and Intron 6, where the parental copy-number-regions with the most CNV occurrences were selected for investigation in the present study. Orange arrowheads and bracket indicate the locations of the two investigated copy-number-regions, *viz.*, nsv1177513 at chr5:160,715,688-160,717,804 with identical CNVs from 226 samples, and esv2730987 at chr5:160,796,703-160,797,020 with three different CNVs from three samples.

*GABRB2* is known to contain a considerable number of CNVs, comprising both CN-gains and CN-losses. Among them, nsv1177513 displays the highest CNV frequency in dbVar with 226 occurrences, while esv2730987 displays 3 occurrences (**Figure 1** and **Supplementary Tables S1A, B**). When combined male and female Chinese SCZ white blood cell DNA samples were analyzed by qPCR and compared to controls (**Table 2**), a two-fold increase of CN-gains was observed in the cases over controls, the frequency in esv2730987 was 24.21% in Chinese SCZ patients, exceeding that of 12.94% in controls with OR = 2.15 and *p* = 5.32E−4 (**Table 2**). For different genders, the frequency in esv2730987 was 26.88% in Chinese male SCZ patients, exceeding that of 16.15% in male controls with OR = 1.91 and *p* = 1.93E−2; and 20.80% in Chinese female SCZ patients, exceeding that of 8.80% in female controls with OR = 2.72 and *p* = 7.55E−3. Even more strikingly, the frequency in nsv1177513 was 54.39% in combined male and female Chinese SCZ patients, exceeding that of 26.57% in controls with OR = 3.29 and *p* = 1.28E−11. Its frequency was 53.13% in Chinese male SCZ patients, exceeding that of 26.09% in male controls with OR = 3.21 and *p* = 7.30E−7; and 56.00% in Chinese female SCZ patients, exceeding that of 27.20% in female controls (OR = 3.41, *p* = 3.85E−6).

**Table 2:** Copy number variations in schizophrenia patients from two ethnic groups.

Ethnic Group	Gender^a^	Control (CNVs)^b^			SCZ (CNVs)^c^			*p*-Value^d^	OR (95% CI)^e^
		**L**	**N**	**G (%)**	**L**	**N**	**G (%)**		
**esv2730987**									
Chinese	M + F	7	242	37 (12.94)	1	215	69 (24.21)	5.32E−04	2.15 (1.39–3.33)
Chinese	M	6	129	26 (16.15)	0	117	43 (26.88)	1.93E−02	1.91 (1.11–3.30)
Chinese	F	1	113	11 (8.80)	1	98	26 (20.80)	7.55E−03	2.72 (1.28–5.79)
German	M + F	8	193	9 (4.29)	7	177	23 (11.11)	8.84E−03	2.79 (1.26–6.19)
German	M	4	101	5 (4.55)	6	87	8 (7.92)	3.08E−01	1.81 (0.57–5.71)
German	F	4	92	4 (4.00)	1	90	15 (14.15)	1.19E−02	3.96 (1.27–12.36)
**nsv1177513**									
Chinese	M + F	31	179	76 (26.57)	10	120	155 (54.39)	1.28E−11	3.29 (2.32–4.68)
Chinese	M	18	101	42 (26.09)	4	71	85 (53.13)	7.30E−07	3.21 (2.01–5.13)
Chinese	F	13	78	34 (27.20)	6	49	70 (56.00)	3.85E−06	3.41 (2.01–5.78)
German	M + F	20	149	41 (19.52)	21	109	77 (37.20)	6.17E−05	2.44 (1.57–3.80)
German	M	7	77	26 (23.64)	3	56	42 (41.58)	5.33E−03	2.3 (1.27–4.16)
German	F	13	72	15 (15.00)	18	53	35 (33.02)	2.57E−03	2.79 (1.41–5.53)

a Gender: M = male, F = female, M + F = male + female.

b Control samples: mean age 24.4 ± 9.2 years in Chinese, and 29.3 ± 9 years in German; L = CN-loss, N = CN-neutral, G = CN-gain.

c SCZ samples: mean age 39.7 ± 12.7 years in Chinese, and 31.3 ± 9.3 years in German.

d p-Value (unadjusted for multiple testing) by Chi-square test between G and L+N of controls vs. G and L+N of SCZ patients.

e Odds ratio = [G/(L+N) in SCZ]/[G/(L+N) in Control]. 95% confidence interval is indicated in the parentheses.

For the German cohorts, the frequency of CN-gains in esv2730987 in the combined male and female SCZ samples was 11.11%, higher than the controls at 4.29% with OR = 2.79 and *p* = 8.84E−3. Its frequency in the male SCZ patients was 7.92%, which was not significantly higher than the male controls at 4.55% with only *p* = 3.08E−1 (OR = 1.81); whereas its frequency in the female SCZ patients was 14.15%, significantly higher than the female controls at 4.00% with OR = 3.96 and *p* = 1.19E−2. Moreover, the frequency of nsv1177513 was 37.20% for the combined male and female German SCZ patients, which was significantly higher than the controls at 19.52% with OR = 2.44 and *p* = 6.17E−5. Its frequency was 41.58% in the male SCZ patients, higher than the male controls at 23.64% with OR = 2.30 and *p* = 5.33E−3; its frequency was 33.02% in the female SCZ patients, higher than the female controls at 15.00% with OR = 2.79 and *p* = 2.57E−3. Therefore, for both the Chinese and German cohorts, female SCZ patients showed higher CN-gain occurrences at both esv2730987 and nsv1177513 compared to female controls. Overall, it was estimated that 9.36% of the variance in heritability of SCZ liability could be explained by carrying a CN-gain within either or both of the two highly recurrent CNVs in *GABRB2* (**Supplementary Table S3**).

Between the two ethnic populations, the frequency of CN-gain in esv2730987 was significantly higher in male Chinese controls compared to male German controls with OR = 4.04 and *p* = 5.91E−3; whereas there was no significant difference between female Chinese and female German controls (**Supplementary Table S4A**). For SCZ-patients, CN-gains in both esv2730987 and nsv1177513 were significantly higher in the Chinese compared to the German cases for the male, female or combined male and female groups, with OR ranging from 1.59 to 4.27 and *p*-values from 2.06E−3 to 2.63E−44 (**Supplementary Table S4B**). No significant difference in CN-gains was observed between the genders in any of the ethnic groups (**Supplementary Table S4C**).

There were significant reductions in CN-losses in Chinese male SCZ patients compared to controls at both esv2730987 (*p* = 4.01E−2) and nsv1177513 (*p* = 4.28E−3). On the other hand, there was no case-control difference at either esv2730987 or nsv1177513 in the Chinese female group or the German male or female group (**Supplementary Table S4D**). CN-losses at nsv1177513 were fewer only in the male German SCZ group compared to the female German SCZ group (*p* = 5.00E−3, **Supplementary Table S4C**). Notably, there were fewer neutral copy numbers in the Chinese and German SCZ groups compared to the controls (**Table 2** and **Supplementary Table S4D**) in agreement with earlier reports (27).

### CNVs in Premenstrual Dysphoric Disorder Cohort of Chinese Origin

When female DNA samples of women with PMDD were compared to those of female non-PMDD controls, the two groups showed a significant difference with respect to the frequencies of CN-gains at both esv2730987 and nsv1177513. The 66.51% frequency in esv2730987 at PMDD patients exceeded that of 15.87% in controls (OR = 10.53, *p* = 4.34E−26), and the 43.72% frequency at nsv1177513 in PMDD patients likewise exceeded that of 24.52% in controls (OR = 2.39, *p* = 3.19E−5). While there was no significant change in CN-losses at either esv273098 or nsv1177513 between PMDD patients and controls, there were far fewer instances of neutral copy numbers at both esv273098 (*p* = 2.49E−33) and nsv1177513 (*p* = 6.08E−12) in PMDD patients compared to the controls (**Table 3** and **Supplementary Table S4E**).

**Table 3:** Copy number variations in PMDD female patients of Chinese origin.

CNV ID^a^	Control (CNVs)^b^				PMDD (CNVs)^c^			*p-Value^d^*	OR (95% CI)^e^
	**L**	**N**	**G**	****	**L**	**N**	**G**		
esv2730987	5	170	33 (15.87)		4	68	143 (66.51)	4.34E−26	10.53 (6.60–16.81)
nsv1177513	5	152	51 (24.52)		4	117	94 (43.72)	3.19E−05	2.39 (1.58–3.62)

a CNV region in dbVar.

b Control samples: mean age 21.4 ± 2.1 years; L = CN-loss, N = CN-neutral, G = CN-gain.

c PMDD samples mean age 24.5 ± 4.1 years.

d p-Value (unadjusted for multiple testing) by Chi-square test between G and L+N of controls vs G and L+N of PMDD patients.

e Odds ratio = [G/(L+N) in PMDD]/[G/(L+N) in Control]. 95% confidence interval is indicated in the parentheses.

### CNV Haplotypes in SCZ and PMDD Cohorts

When the frequencies of the nine haplotypes based on the gain (G), neutral (N), or loss (L) at the esv2730987 and nsv1177513 loci were compared between the SCZ or PMDD patients and their controls, the frequency of the G-G haplotype (*viz.*, CN-gain at both esv2730987 and nsv1177513) showed the largest increase in both SCZ and PMDD patients relative to their controls, yielding ORs of 2.73 and 3.89 in the combined-gender groups of the Chinese and German SCZ cohorts, respectively, and OR of 5.81 in the PMDD cohort (**Table 4**). The OR between patients and controls for the G-G double-gain at these two CNV loci reached 6.78 in the German female SCZ group, exceeding that of 3.96 and 2.79 for the individual esv2730987 and nsv1177513 locus (**Table 2** and **Supplementary Table S4F**). However, in other case-control comparisons, the ORs for the double-gain haplotype was between that for the gain at esv2730987 and the nsv1177513 locus (**Supplementary Table S4F**). Another haplotype showing significant increase in SCZ patients relative to controls was the N-G haplotype in both the Chinese combined-gender group (OR = 2.36, *p* = 1.68E−5) and the German combined-gender group (OR = 1.87, *p* = 1.19E−2). The G-N haplotype also occurred more in the PMDD patients compared to controls reaching an OR of 5.57 (*p* = 5.26E−11). The N-N haplotype frequencies were all significantly reduced in patients compared to controls for both diseases throughout the different population or gender groupings (**Supplementary Table S4F**).

**Table 4:** Haplotypes of copy number variations in SCZ and PMDD cohorts.

Haplotype ^a^	No. of Cases		No. of Controls		*p*-Value ^b^	OR (95% CI) ^c^
	With Haplotype (%)	Without Haplotype	With Haplotype (%)	Without Haplotype		
**Chinese SCZ cohort (Male + Female)**							
G-G	57 (20.00)	228	24 (8.39)	262	1.16E−04	2.73 (1.64–4.54)
G-N	12 (4.21)	273	13 (4.55)	273	1.00E+00	0.92 (0.41–2.06)
L-L	1 (0.35)	284	3 (1.05)	283	6.18E−01	0.33 (0.03–3.21)
L-N	0 (0.00)	285	4 (1.40)	282	1.33E−01	0.00 (0.00–NaN)
N-G	98 (34.39)	187	52 (18.18)	234	1.68E−05	2.36 (1.60–3.47)
N-L	9 (3.16)	276	28 (9.79)	258	2.30E−03	0.30 (0.14–0.65)
N-N	108 (37.89)	177	162 (56.64)	124	1.07E−05	0.47 (0.33–0.65)
						
**German SCZ cohort (Male + Female)**							
G-G	18 (8.70)	189	5 (2.39)	204	9.37E−03	3.89 (1.41–10.67)
G-L	1 (0.48)	206	0 (0.00)	209	9.96E−01	Inf (NaN–Inf)
G-N	4 (1.93)	203	4 (1.91)	205	1.00E+00	1.01 (0.25–4.09)
L-G	1 (0.48)	206	0 (0.00)	209	9.96E−01	Inf (NaN–Inf)
L-L	2 (0.97)	205	4 (1.91)	205	6.90E−01	0.50 (0.09–2.76)
L-N	4 (1.93)	203	4 (1.91)	205	1.00E+00	1.01 (0.25–4.09)
N-G	58 (28.02)	149	36 (17.22)	173	1.19E−02	1.87 (1.17–2.99)
N-L	18 (8.70)	189	16 (7.66)	193	8.35E−01	1.15 (0.57–2.32)
N-N	101 (48.79)	106	140 (66.99)	69	2.53E−04	0.47 (0.32–0.70)
						
**Chinese PMDD cohort (Female)**							
G-G	60 (27.91)	155	13 (6.25)	195	8.21E−09	5.81 (3.08–10.96)
G-L	3 (1.40)	212	0 (0.00)	208	2.58E−01	Inf (NaN–Inf)
G-N	80 (37.21)	135	20 (9.62)	188	5.26E−11	5.57 (3.25–9.54)
L-G	3 (1.40)	212	0 (0.00)	208	2.58E−01	Inf (NaN–Inf)
L-L	0 (0.00)	215	2 (0.96)	206	4.64E−01	0.00 (0.00–NaN)
L-N	1 (0.47)	214	3 (1.44)	205	5.92E−01	0.32 (0.03–3.09)
N-G	31 (14.42)	184	38 (18.27)	170	3.47E−01	0.75 (0.45–1.27)
N-L	1 (0.47)	214	3 (1.44)	205	5.92E−01	0.32 (0.03–3.09)
N-N	36 (16.74)	179	129 (62.02)	79	3.58E−21	0.12 (0.08–0.19)

a Haplotype based on the gain (G), neutral (N), and loss (L) at the esv2730987 and nsv1177513 loci. The G, N or L before hyphen indicates CNV status at esv2730987 and that after hyphen the status at nsv1177513. Only seven of the nine possible haplotypes were found in the Chinese SCZ cohort.

b p-Value (unadjusted for multiple testing) by Chi-square test for numbers of samples with haplotype and without haplotype among cases vs those among controls.

c OR = (No. of cases with haplotype/No. of cases without haplotype)/(No. of controls with haplotype/No. of controls without haplotype). 95% confidence interval is indicated in the parentheses.

## Discussion

Previously, a range of rare, high-penetrant CNVs have been identified at various locations in the human genome that increased the risk of SCZ as well as other psychiatric diseases such as mental retardation and autism (28). Rare CNVs at 1q21.1, 2p16.3 (NRXN1), 3q29, 7q11.2, 15q13.3, distal 16p11.2, proximal 16p11.2, and 22q11.2 conferred significant risk of SCZ with ORs of 2-60, accounting for 0.85% of SCZ cases (29, 30). More generally, we have found that recurrent germline CNV profiles predict cancer susceptibility (31). Therefore, the present study was directly to recurrent rather than rare CNVs.

### Highly Recurrent CNVs in *GABRB2* Associated With SCZ

The *GABRB2* gene, SNPs in which associated with SCZ in Chinese with an OR of 2.02 to 2.50 (3), harbored a number of recurrent germline CNVs including esv2730987 and nsv1177513, the latter of which has the highest frequency recorded in *GABRB2*. Results showed in **Table 2** indicated that the combined male-female frequencies for CN-gains in esv2730987 and nsv1177513 were both significantly higher in cases over controls, establishing the associations of highly recurrent CNVs with SCZ, in both Chinese and German. Based on the results from this study, it is estimated that 9.36% of the variance in SCZ liability can be explained by carrying a CN-gain within either or both of the two highly recurrent CNVs in *GABRB2* (**Supplementary Table S3**). In contrast, a previous study has reported that only about 2.5-3% of SCZ heritability can be explained collectively by a total of 32 SCZ-associated SNPs and CNVs (26).

The *p*-values were more significant for CN-gain increases in both nsv1177513 and esv2730987 in the Chinese relative to the German SCZ patients. With respect to genders, the CN-gains in both esv2730987 and nsv1177513 were significantly elevated in both female Chinese and female German patients, as well as the male Chinese patients. In contrast, the German male patients displayed significant elevation in CN-gains in nsv1177513 but not in esv2730987. This weaker correlation of CN-gains with SCZ in esv2730987 in the male German patients was also evident in the weaker correlation of the CN-gains in the nsv1177513 in the male German patients and in the esv2730987 in the female Chinese patients, but opposite for the stronger correlation observed in nsv1177513 in the male Chinese patients compared to the female ones.

Previously, with the rare high-penetrant CNVs that increased the risk of SCZ, autism and mental retardation, disease propensity was only associated with both CN-gains and CN-losses (28), in contrast to the association of CN-gains with SCZ in esv2730987 or nsv1177513. The nature of the perturbations occasioned by CNVs could be multifaceted and vary from one gene to another. Earlier, pairwise co-localizations of 42 genomic features have enabled a tripartite division of the human genomic sequence into the Genic, Proximal, and Distal sequence zones, which are distinguishable in terms of their constituent genomic variants (32). The DNA sequences in Proximal zones, where *GABRB2* locate, tolerate small genetic variants such as microsatellites but not long genetic variants such as large CNVs, possibly on account of excessive disruption of inter-gene distances involving the gene-regulatory elements by the long variants (32). The mechanisms of the disruption by CN-gains in either esv2730987 or nsv1177513 yet remain to be elucidated. Nevertheless, the strong association and high heritability of liability demonstrated in this study support that the two recurrent CNVs in *GABRB2* may serve as excellent biomarkers.

### Association of Highly Recurrent CNVs With PMDD

Neuropsychiatric changes in PMDD have been observed and characterized extensively (19, 33, 34). Women with PMDD often present recurrent symptoms of anxiety and depression, which have been associated with genetic variations of *ESR1* (18). Studies have linked progesterone to modulation of the medial prefrontal cortex (mPFC) and testosterone to the orbitofrontal cortex (OFC), along with the implication of various cortical and subcortical regions in an attempt to explain the psychiatric vulnerability in women (35). In addition, other studies have shown that OFC function varies with changes in progesterone concentration throughout the menstrual cycle, suggesting a relationship between psychiatric effect of PMDD with the coupling of mPFC and OFC with the amygdala (34). Moreover, PMDD also has been linked to the impaired activation of the left amygdala and dorsal anterior cingulate gyrus during emotional processing (36–38).

In another approach, it has been reported that the estrogen-sensitive epigenetic ESC/E(Z) complex in lymphoblastoid cell lines (LCLs) differed between women with PMDD and non-affected female controls at the RNA and protein levels (19), pointing to a linkage between periods of neurosteroid fluctuation and altered sensitivity of GABA_A_ receptors to neurosteroids (16, 17, 39). The present finding that recurrent CN-gains at nsv1177513 and esv2730987 were enriched in PMDD subjects thus expanded further the spectrum of neuropsychiatric disorders associated with *GABRB2* beyond SCZ (3, 8), bipolar disorder (10), and heroin addiction (20), and provided the first known instance of PMDD association with a gene expressed in the central nervous system.

A striking parallel between PMDD and SCZ pertaining to their associations with the recurrent CNVs in *GABRB2* was that the double CN-neutral haplotype N-N at the two CNV sites was protective against both diseases. Whereas the double CN-gain haplotype G-G was risk-conferring to both diseases. However, disease-association was stronger at esv2730987 in PMDD, but stronger at nsv1177513 in SCZ. More significant associations were observed with PMDD compared to SCZ with respect to both genotypes (**Tables 2** and **3**) and haplotypes (**Table 4**).

### Recurrent CNVs as Markers in Association Studies

The recurrent CNVs represent useful markers for the search of genetic components attributable to complex disorders. The association of recurrent CNVs with complex disorders have been revealed by their large ORs reaching up to 3.96 in the SCZ cohorts and 10.53 in the PMDD cohorts in the present study, as well as by the previous finding of useful recurrent CNVs for cancer predisposition (31). Different from the frequently used common SNPs and rare structural variations in association studies, the recurrent CNVs represent the intermediate form of genetic variants. On the one hand, they are more robust than SNPs regarding the confounding effects brought about by complex genetic forces like recombination and natural selection. On the other hand, due to their high recurrency, they could readily explain more of the disease variations in the population than rare structural variations do. Therefore, in-depth investigation of recurrent CNVs is needed for future association studies of complex diseases and traits.

In addition to the recurrent CNVs in *GABRB2*, SCZ has also been associated with SNPs in the same gene, underlining the central role of this gene in the SCZ etiology as well as the genetic instability of the gene. Such instability would enhance the occurrences of genetic variants, in both common, e.g., SNPs and rare, e.g., CNVs forms. Given the functional significance of *GABRB2* in the central nervous system, sequence and structural alterations could lead to functional perturbations of the gene, and thereby associations with psychiatric disorders. On the other hand, the associations of PMDD also with the same recurrent CN-gains in *GABRB2* helped to explain the symptomology of PMDD involving not only pain but also emotional disturbance, and pointed to possible overlap between the etiological mechanism for SCZ and PMDD.

Although, close association between *GABRB2* and SCZ has been confirmed by meta-analyses of candidate genes for SCZ (40, 41), genome-wide association studies (GWASs) have not reported to date any significant association between genetic variants in *GABRB2* and SCZ (42, 43). The GWAS results might seem inconsistent with the positive signals from the genome-wide linkage and candidate gene studies, as well as the SCZ-like phenotypic alterations displayed by the *GABRB2* knock-out mice (13). However, a fine-resolution linkage disequilibrium analysis of a 3,551-bp segment of *GABRB2* revealed active recombination together with intense positive selection on the derived alleles in this region (7). Such co-occurrence of recombination and positive selection could blur the local SNP-based signals in GWAS, causing the genetic components attributable to complex traits and diseases difficult to recognize, whereas CNV-based signal could be more resistant to such confounding factors. Therefore, genes and genomic regions subject to both active recombination and selection would merit close examination and require the employment of markers, such as CNVs, that are more robust to the effects of recombination-selection co-occurrence to avoid any missing heritability pertaining to the development of complex diseases.

### Closing Remarks

With the employment of recurrent CNV markers, the present study has established for the first time the genetic associations between CN-gains in *GABRB2* and two different psychiatric disorders, namely SCZ and PMDD in face of the effects of active recombination and natural selection on this gene. Most neuropsychiatric disorders have significant genetic components. Different neuropsychiatric disorders are often related to one another in their phenotypic characteristics, which could stem out of common genetic variations, or “shared genetics”, exemplified by the genetic overlap between Alzheimer’s disease and bipolar disorder with respect to the *MARK2* and *VAC14* genes (44), the genetic overlap between SCZ, bipolar disorder, and intelligence (45), and the shared genetic variants between SCZ and lung cancer (46). In the same view, the present finding of the same set of recurrent CN-gains in *GABRB2* associated two clinically very different psychiatric disorders delineate further the central role of this gene in neuropsychiatric disorders, and providing useful insights into overlapping genetic mechanisms underlying the comorbidities of different psychiatric disorders.

## Data Availability Statement

The original contributions presented in the study are publicly available. This data can be found at https://www.ncbi.nlm.nih.gov/clinvar/, under the accession numbers: SCV001334130, SCV001334131, SCV001334132, SCV001334133, SCV001334134, SCV001334135, SCV001334136, SCV001334137.

## Ethics Statement

The studies involving human participants were reviewed and approved by Beijing Hui Long Guan Hospital Hong Kong Red Cross. The hospitals affiliated with the University of Wurzburg German Shandong University of Traditional Chinese Medicine Hospital, Jinan, People’s Republic of China. The patients/participants provided their written informed consent to participate in this study.

## Author Contributions

HX conceived and designed the experiments. AU, W-KM, XLo, and MK performed the experiments and analysis of the data. TH identified the two recurrent CNV regions from database and designed the primers. LH and XZ coordinated the collection of schizophrenia samples of Chinese origin. PS, MG, JW, HW, XLi, WS, and MQ coordinated the collection of PMDD and control cohorts, and AU, XLo, and HX wrote the paper.

## Funding

The study was supported by grants to HX from University Grants Council (SRF116SC01, UROP18SC06, and UROP20SC07) and Innovation Technology Council (ITS113/15FP and ITT/026/18GP) of Hong Kong SAR; Shenzhen Municipal Council of Science and Technology, Guangdong (SZ-SZST11808); Shandong Province First Class Disciple Development Grant and Tai-Shan Scholar Program, Shandong; and Ministry of Science and Technology (National Science and Technology Major Project, No. 2017ZX09301064), People’s Republic of China, as well as grants from National Natural Science Foundation of China to MQ (No. 8157151623) and JW, respectively (No. 81603510).

## Conflict of Interest

The authors declare that the research was conducted in the absence of any commercial or financial relationships that could be construed as a potential conflict of interest.
